# Mapping of brain tissue hematocrit in glioma and acute stroke using a dual autoradiography approach

**DOI:** 10.1038/s41598-018-28082-w

**Published:** 2018-06-29

**Authors:** A. Broisat, B. Lemasson, M. Ahmadi, N. Collomb, S. Bacot, A. Soubies, D. Fagret, C. Rémy, C. Ghezzi, E. L. Barbier

**Affiliations:** 1 Univ. Grenoble Alpes, Inserm, CHU Grenoble Alpes, U1039, LRB, F-38000 Grenoble, France; 2 Univ. Grenoble Alpes, Inserm, U1216, GIN, F-38000 Grenoble, France; 3 Univ. Grenoble Alpes, Inserm, CHU Grenoble Alpes, IRMaGe, CNRS, F-38000 Grenoble, France

## Abstract

Hematocrit (Hct) determines the ability of blood to carry oxygen. While changes in systemic Hct are known to impact stroke or tumor control, changes in local (tissue) Hct (tHct) induced by these diseases have however received little attention. In this study, we evaluate tHct in acute stroke and in glioma models using a new approach to map tHct across the brain, a dual isotope autoradiography, based on injections of ^125^I-labeled albumin and ^99m^Tc-lalbeled red blood cells in the same animal. For validation purpose, tHct was mapped in the rat brain (i) under physiological conditions, (ii) following erythropoietin injection, and (iii) following hemodilution. Then, tHct was then mapped in stroke (middle cerebral artery occlusion) and tumor models (9LGS and C6). The mean tHct values observed in healthy brains (tHct = 29 ± 1.3%), were modified as expected by erythropoietin (tHct = 36.7 ± 2.6%) and hemodilution (tHct = 24.2 ± 2.4%). Using the proposed method, we observed a local reduction, spatially heterogeneous, in tHct following acute stroke (tHct = 19.5 ± 2.5%) and in both glioma models (9LGS: tHct = 18.5 ± 2.3%, C6: tHct = 16.1 ± 1.2%). This reduction and this heterogeneity in tHct observed in stroke and glioma raises methodological issues in perfusion imaging techniques where tHct is generally overlooked and could impact therapeutic strategies.

## Introduction

The volume fraction of red blood cells in blood, hematocrit (Hct), can be measured during a simple blood test and serves as an indicator of health status. It varies across subjects (age, sex) and over time^1^, but also within the vascular system. For example, Hct varies by about 20% across the different regions of a healthy rat brain^2,3^. Moreover, Hct is 20 to 40% lower in capillaries than in major arteries^2,3^ due to the Fahraeus effect^4,5^. These local variations of Hct represent the local variations of the ability of blood to carry oxygen to the tissue. Mapping tissue Hct (tHct) is thus of interest to evaluate the local status of Hct as well as the intralesional heterogeneity. While change in systemic Hct are known to impact stroke^6^ or tumor control^7^, intralesional Hct heterogeneity induced by these diseases have however received little attention. In preclinical studies, this lack of interest may originate from a lack of available method to map tHct in a single animal.

To map tHct, autoradiography provides high-resolution, quantitative, *post mortem* imaging of radiolabeled compound distribution in tissue sections. At a preclinical level, high spatial resolution is required to detect small tissue structures or small lesions and to assess the intralesional heterogeneity. Such spatial resolution may not be achieved with preclinical *in vivo* nuclear imaging. Autoradiographic quantification of brain tHct has first been described in the 1950s using radiolabeled albumin or red blood cells (RBC) for the determination of plasmatic distribution volume (Vp) or RBC distribution volume (Vrbc), respectively. Due to the low sensitivity of x-ray films, long half-life isotopes such as ^131^I (half-life: 8.0 days) or ^125^I (59.4 days) for the albumin and ^51^Cr (27.7 days), ^59^Fe (44.5 days) or ^55^Fe (2.7 years) for RBC were employed, thereby allowing long exposure times of several weeks. With these long half-life isotopes, Vp and Vrbc had to be obtained on separated groups of animals, and average tHct were derived in a given brain structure. It was therefore not possible to map Hct in the lesion from a single animal using autoradiography. Nowadays, high sensitivity autoradiography modalities, such as phosphor-imager, allow imaging short half-life isotopes. Therefore, one can now perform dual isotope imaging within the same tissue sections, using two isotopes with distinct half-life and sequential or dynamic exposures.

In this study, we propose a new approach to map the tHct across the brain: a dual isotope autoradiography, using ^125^I-labeled albumin (59.4 days half-life) and ^99m^Tc-labeled RBC (6 hours half-life). To validate this new approach, tHct was mapped in the rat brain under physiological conditions as well as following a decrease in systemic Hct (hemodilution) or following an increase in systemic Hct (erythropoietin (EPO) injection). Finally, we evaluated the change of local Hct in rat models of tumor (two glioma models) and of stroke (middle cerebral artery occlusion).

## Material and Methods

### Animal preparation

All animal procedures conformed to French government guidelines and were performed under permit 380820 and B3851610008 (for experimental and animal care facilities) from the French Ministry of Agriculture (Articles R214-117 to R214-127 published on 7 February 2013). This study is in compliance with the ARRIVE guidelines (Animal Research: Reporting *in Vivo* Experiments)^8^ with the approval of the “Grenoble Institut des Neurosciences” ethical committee (National agreement n°004). Male rats aged of 7 weeks (Charles River, France) were housed in groups of 3-4 in Plexiglas cages under standard laboratory condition (12 h light/dark cycle with lights off at 7:00 p.m. and controlled temperature in 22 ± 2 °C). Water and standard laboratory chow were provided *ad libitum*. For all experiments, rectal temperature was monitored and rats were maintained at 37.0 ± 0.5 °C. Anesthesia was induced by the inhalation of 5% isoflurane (Abbott Scandinavia AB, Solna, Sweden), and maintained throughout all surgical and imaging procedures with 2–2.5% isoflurane through a facial mask in 80% air-20% oxygen. All animals were imaged by MRI (see MR imaging subsection) and two hours after underwent the nuclear imaging protocol (see Mapping of brain hematocrit subsection), except animals from for the Hemodilution group (see below), which were imaged by nuclear imaging only. Six groups of animals were considered:

#### Control group

Wistar rats (n = 10) were used as controls. Two animals were used as blood donor for RBC labeling. The control group is therefore composed of 8 Wistar animals (268 ± 23 g).

#### EPO group

Wistar rats (n = 11) were used in the EPO group. Animals received an injection of EPO (5000 U/kg IV) one week prior to the experiment. Two animals were used as blood donor for RBC labeling and 1 animal died during the MRI protocol. The EPO group is therefore composed of 8 animals (252 ± 26 g).

#### Hemodilution group

Wistar rats (n = 10) were used in the Hemodilution group. On the day of the experiment, isovolemic hemodilution was performed by replacing 30% of the blood (2% of body weight, ≈5 ml) by the same volume of saline (NaCl 0.9% containing 5% of serum albumin) 15 minutes prior to the administration of radiotracers. For this purpose, arterial and venous catheters were inserted in the femoral artery and vein of anesthetized animals for either blood drawing and saline infusion (1 ml.min^−1^), respectively. Two animals were used as blood donor for RBC labeling. The Hemodilution group is therefore composed of 8 animals (291 ± 20 g).

#### Glioma groups (9LGS and C6)

Bupivacaine (8 mg.kg^−1^; Centravet, France) was subcutaneously injected before incision to prevent postoperative pain. Tumor cell inoculation was performed into the right caudate nucleus (coordinates from bregma: Anterior – Posterior = 0, Medial – Lateral = 3, Dorsal – Ventral = 5.5 mm). After injection, the burr hole was filled, the skin incision sewed and rats revived in an incubator before returning to the animal facility. The two tumor models were performed as described below. 9LGS cells (ATCC, American Type Culture Collection) were implanted in the brain of Fisher 344 rats (n = 14). One µl of cell suspension in serum-free RPMI1640 medium containing 1.10^4^ cells was inoculated and experiments were performed 10 days after. Two animals were used as blood donor for RBC labeling and 4 animals died during the surgery or during the MRI protocol. The 9LGS group is therefore composed of 8 animals (223 ± 8 g). C6 cells (ATCC, American Type Culture Collection) were implanted in the brain of Wistar rats (n = 11). Five µl of cell suspension in serum-free RPMI1640 medium containing 1.10^5^ cells were inoculated and experiments were performed 20 days after. Two animals were used as blood donor for RBC labeling and 1 animal died during the surgery. The C6 group is therefore composed of 8 animals (237 ± 8 g).

#### Stroke group

Sprague Dawley rats (n = 13) underwent a permanent focal brain ischemia induced by intraluminal occlusion of the right middle cerebral artery^9^. Briefly, the right carotid arterial tree was isolated. A cylinder of melted adhesive (length 2 mm; diameter 0.38 mm) attached to a nylon thread (diameter 0.22 mm) was advanced from the lumen of the external carotid artery into the internal carotid artery up to 5 mm after the external skull base. Rats were imaged by MRI 60 min and by autoradiography 120 min after the occlusion. Two animals without surgery were used as blood donor for RBC labeling and 4 animals died during the surgery. The Stroke group is therefore composed of 7 animals (364 ± 19 g).

### MR imaging

To delineate brain structures and brain lesion, anatomical T_2_-weighted were acquired with a horizontal bore 4.7 T Biospec animal imager (Bruker Biospin). For animals in Stroke and Glioma-groups, Apparent Diffusion Coefficient (ADC) was mapped using diffusion-weighted imaging (cf. Supplementary Information).

### Mapping of brain hematocrit

#### RBC labeling

^99m^Tc-labeled red blood cells (^99m^Tc-RBCs) were prepared with the commercial TechneScan PYP kit (Mallinckrodt). Radiolabeled RBCs *in vitro* stability was evaluated by centrifugation at 6 h and *in vivo* stability was determined by measuring RBC activity following euthanasia (cf. Supplementary Information).

#### Plasma labeling

Radiolabeling of Bovine Serum Albumin (BSA) with ^125^I was prepared as previously described by Salacinski *et al*.^10^. *In vivo* stability was also determined by measuring plasma activity following euthanasia (cf. Supplementary Information).

#### Experimental design

A 0.2 ml mixture of ^99m^Tc-RBC (45.2 ± 9.1 MBq) and ^125^I-BSA (3.9 ± 0.6 MBq) was injected in the saphenous vein. Following a 15 minutes equilibration period^11^, a sample of blood was taken from the myocardium and the animals were then euthanized by rapid heart excision. The brain was then immediately harvested and frozen into −40 °C isopentane, together with reference tissue samples of liver, salivary gland and muscle. Total blood hematocrit (bHct) was determined using capillary tube centrifugation using the myocardium blood sample (Jouan hema-c, France).

#### Gamma-well counting (GWC)

Samples of blood, reference tissues and brain were weighed and ^99m^Tc-RBC and ^125^I-BSA activity was determined using a gamma-well counter (Wizard2, Perkin). Activity is expressed as MBq.g^−1^ or as a percent of the injected dose per gram (%ID.g^−1^).

#### Autoradiography

**Figure 1:**
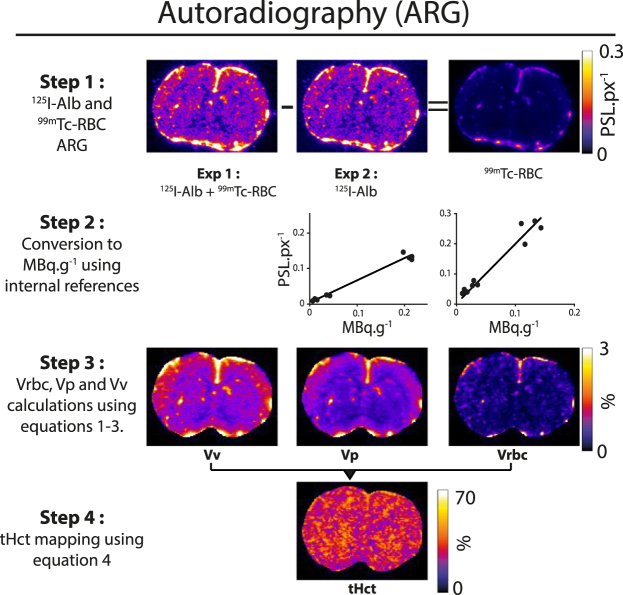
Protocol to map tissue Hct (tHct) using the proposed dual-isotope autoradiography. PSL.px^−1^: PhotoStimulated Luminescence.pixel^−1^.

One hundred microns thick coronal brain cryosections were obtained at the level of the striatum. Reference tissue samples, one drop of pure ^125^I, and diluted blood (1:50 in optimal cutting temperature medium, Tissue-Tek) cryosections were also obtained as internal references (cf. below; Supp. Fig. 1). Autoradiography was performed using a phosphor imager (Fuji BAS-5000). A first overnight exposure was performed to obtain an autoradiography representing the sum of ^99m^Tc-RBC and ^125^I-BSA activities (Fig. 1, step 1: *Exp1*). One week later, a second, one-week, exposure was then performed. Due to the decay of the short half-life emitter ^99m^Tc, the second autoradiography represented the distribution of ^125^I-BSA only (Fig. 1, step 1: *Exp2*).

#### Image analysis

Image analysis was performed using Image-J software^12^ (Fig. 1). *Exp2* intensity was corrected to *Exp1* using the signal from the pure ^125^I area (Supp. Fig. 1), the slices were then co-registered using an automated algorithm^13^ and *Exp2* was subtracted to *Exp1* to obtain maps of the distribution of ^99m^Tc-RBC only (Fig. 1, step 1). Autoradiographic images of brain slices were then converted into MBq.g^−1^ and RBC distribution volume (Vrbc), plasma distribution volume (Vp) and vascular volume (Vv) images were then derived (cf. Supplementary Information).

### Data analysis

For each animal, Vv image was coregistered to the anatomical MRI image using Matlab 2016a (elastic registration; Supp. Fig. 2). The transformation matrix was then applied to Vrbc, Vp and tHct images. For the Hemo group, an anatomical template was used.

Brain from the Control, EPO and Hemodilution groups were automatically segmented into 3 Regions of interest: striatum, cortex, and white matter using a 3D MRI rat brain atlas^14^ and the statistical parametric mapping software (SPM12). For the C6 and 9LGS animals, the tumor region was manually delineated on the anatomical MRI images. For the stroke group, the ischemic lesion was manually delineated as the hypointense area on ADC maps and excluding ventricles and/or small bleeds^15^. The striatum region was manually delineated in the contralateral hemisphere for all pathological animals.

### Statistical analysis

All statistical analysis were conducted with GraphPad Prism software. Two-way ANOVA was used to compare the results obtained in Control, EPO, and Hemodilution groups for all organs, the results obtained in Control, EPO, and Hemodilution groups for each brain region, and the results obtained in each brain region for the three groups. Pearson correlation coefficient was used to characterize the relation between ARG, GWC, and Capillary estimates of bHct as well as the relation between GWC and ARG estimates of Vp, Vrbc, Vv, and Hct. Lesion and contralateral striatum were compared using paired t-tests. Results are expressed as mean ± SD.

### Data availability statement

Most data generated during this study are included in this published article and its Supplementary Information files. The datasets generated during and/or analyzed during the current study are available from the corresponding author on reasonable request.

## Results

For RBC and albumin, the labeling yield was 80 ± 10% and 91 ± 5%, respectively. *In vitro*, 6 hours after labeling, ^99m^Tc radioactivity in supernatant had drift from less than 2% to 2.4 ± 0.8%, confirming a good stability of ^99m^Tc-labeled RBCs. ^125^I-BSA remained stable over a 6 h period after radiolabeling with a radiochemical purity of 95%. *In vivo*, activities of ^99m^Tc and of ^125^I were specific to RBC (99 ± 1%) and plasma (95 ± 2%), respectively.

**Figure 2:**
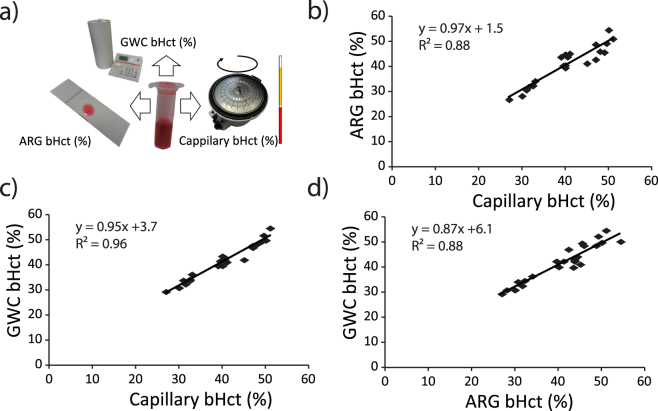
(**a**) Illustration of the 3 methods (gamma-well counting: GWC; dual-isotope autoradiography: ARG and the capillary) used to calculate the Blood Hct (bHct) obtained for each of the 24 animals included in the Control, Hemodilution and EPO groups. Scatter plots of the correlation between (**b)** ARG bHct and Capillary bHct, (**c)** GWC bHct and Capillary bHct and (**d)** GWC bHct and ARG bHct.

The agreement between the 3 methods used to determine bHct was excellent. The Pearson correlation coefficient was very high (R² > 0.88) with a slope close to unity (>0.87) (p < 0.0001 for all three comparisons) (Fig. 2). Despite the fact that the proposed approach appears slightly noisier than the reference capillary methods, these results highlights the ability of dual-isotope autoradiography to obtain accurate estimates of bHct (slope = 0.97).

**Figure 3:**
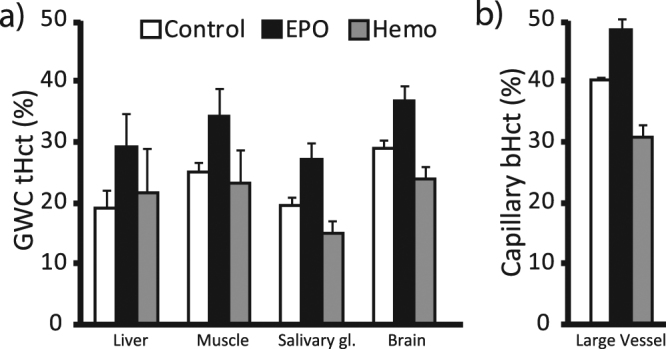
Blood Hct (bHct) estimated across organs using (**a)** the gamma-well counting (GWC) or (**b)** the capillary technique for the Control, Hemodilution and EPO groups. Mean ± SD.

In the four tissues evaluated and in blood, the mean Hct of Control group, determined using GWC, was 42.2 ± 11.8% lower than bHct (Fig. 3; Supp. Table 1). In the Control group, the smallest Hct decrease with respect to large vessels was observed in the brain (from 40.1 ± 0.6% to 29.0 ± 1.3%, a reduction of −27.7 ± 2.9%, p < 0.0001) and the highest Hct decreases were observed in the liver and in the salivary gland (a reduction of −52.4 ± 7.3 and −51.0 ± 3.3%, respectively; p < 0.0001). As expected, administration of EPO increased Hct in all organs (+35.2 ± 12.3%, p < 0.0001), and hemodilution decreased Hct (−14.5 ± 10.7%) in the salivary gland, brain and blood with respect to the control group but not in liver and in muscle where it remained stable following hemodilution. However, the ratios between organ and blood Hct were increased following both EPO and hemodilution (except salivary gland following hemodilution) indicating that the organ responses to these challenges differed from that of bHct.

**Figure 4:**
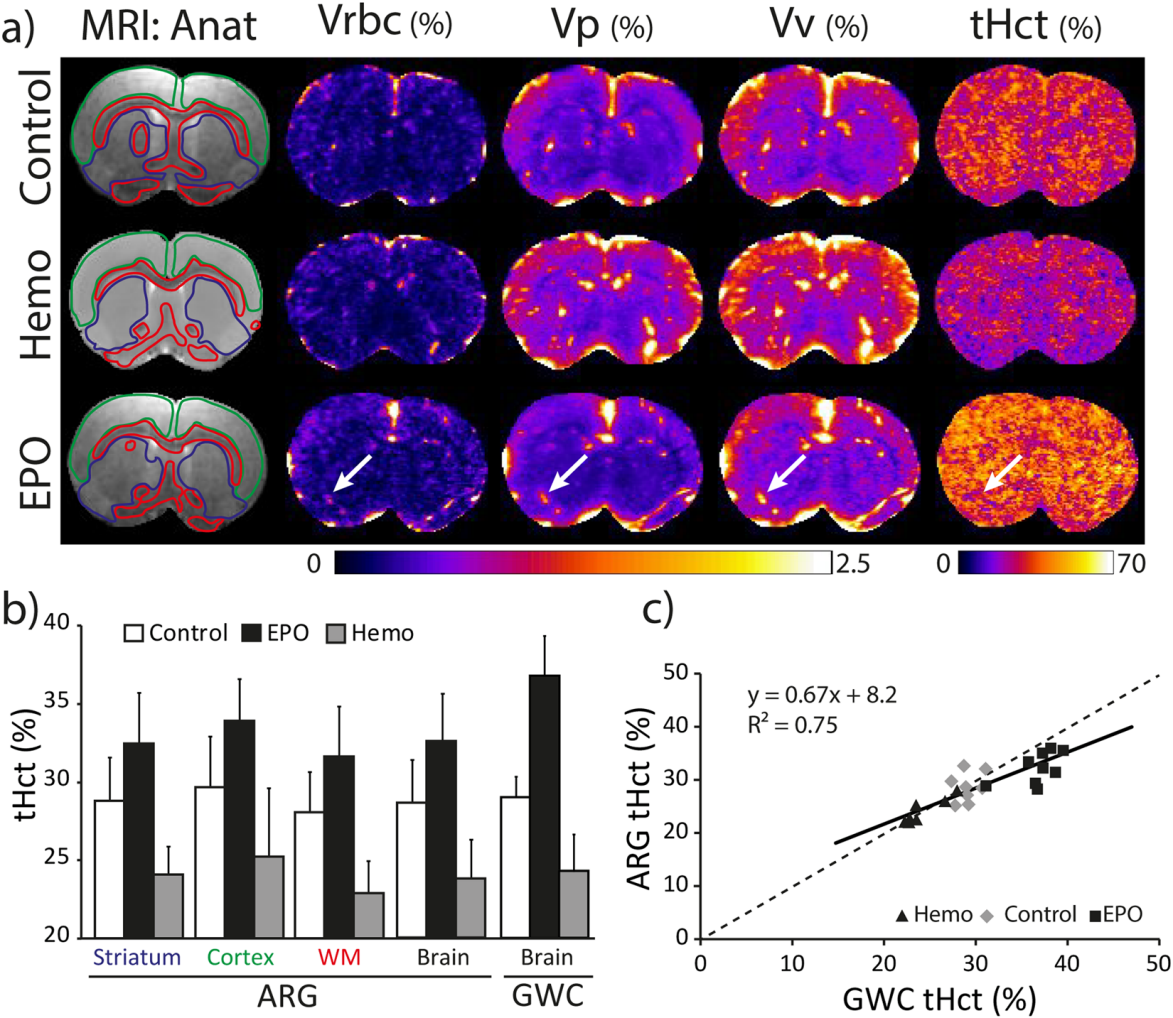
(**a**) Representative images of MRI anatomical images and of red blood cells (Vrbc), plasmatic (Vp), vascular volume (Vv) and tissue hematocrit (tHct) maps obtained by autoradiography for the Control, Hemodilution and EPO groups. Note that the MRI anatomical image for the Hemodilution group is that of a template. For each animal, all images are coregistered. The green, red and blue lines illustrate the automatic delineation of the cortex, white matter and striatum region of interest, respectively. The white arrows point towards a large vessel observed across each ARG maps. (**b**) tHct values measured in each region of interest using the autoradiography (ARG) or the gamma-well counting (GWC) approach. Mean ± SD. (**c**) Scatter plot of the correlation between the tHct values obtained using the ARG and the GWC techniques. Data from the Hemodilution (triangle), Control (diamond) and EPO (square) are pooled (one symbol represents one animal).

In this study, imaging was performed on brain slices. Figure 4a shows the anatomical MRI and ARG images for one animal per group (Control, Hemo and EPO groups). Images from all animals (Supp. Figs 3–5) suggest excellent reproducibility. ARG images are composed of the two raw maps (Vrbc and Vp) and the two maps computed with Eqs [3,4] (Vv and tHct) for the three groups. Large vessels appear as bright spots (Fig. 4a-white arrows) in Vrbc, Vp, and Vv maps but not in the tHct map, where large vessels can barely be distinguished from the surrounding tissue. One can observe than Vrbc, Vp, and Vv exhibit higher values in the cortex than in the striatum, a pattern not observed for tHct (Fig. 4b). The decrease and increase in tHct induced by hemodilution and EPO are readily visible in the corresponding tHct maps (Fig. 4a). These changes in tHct were also detected by GWC, an approach that correlated well with ARG (Fig. 4c). The spatial analysis of brain tHct maps performed with 4 regions indicate that striatum, cortex, and white matter had similar tHct and respond similarly to EPO (a 13.9% increase, p < 0.05) and hemodilution (a 16.3% decrease, p < 0.01) (Fig. 4b; Suppl. Table 2 for the values).

**Figure 5:**
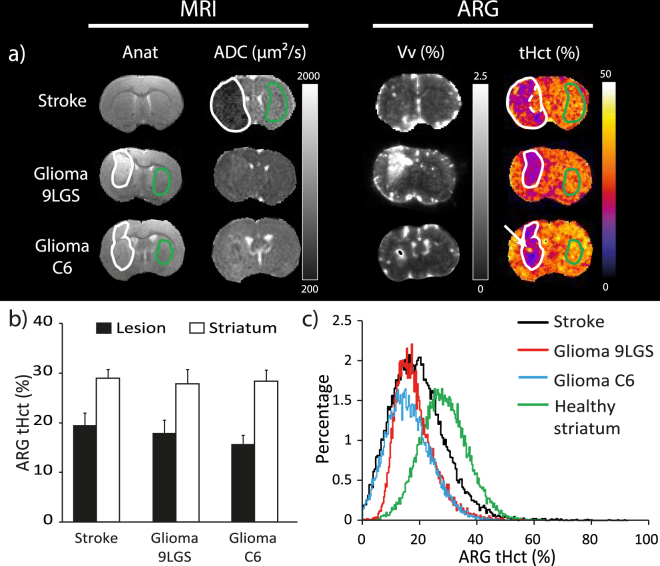
(**a**) Representative images of anatomical images, diffusion (ADC), obtained by MRI, as well as, the vascular volume (Vv) and tissue hematocrit (tHct) maps, obtained by autoradiography, for the Stroke, C6 and 9LGS gliomas. All images are co-registered per animal. The white, green and dashed lines represent the lesion, contralateral and normal appearing areas. The white arrow represents a tHct hotspot inside the C6 glioma. (**b**) tHct values measured using the autoradiography (ARG) approach in lesion or in the healthy striatum for each pathological model (Stroke, 9LGS and C6 gliomas). (**c**) Histogram of tHct values measured by ARG in the stroke (black line), glioma 9LGS (red), glioma C6 (blue line) and Healthy striatum (green line; data from the 3 pathological groups were pooled) areas. Values are displayed as Mean ± SD.

Stroke and gliomas had a strong impact on tHct (Fig. 5). All lesions led to a marked reduction in tHct whereas the contralateral striatum exhibited similar values across stroke, C6, 9LGS, and Control groups (18.0 ± 2.4 vs 28.5 ± 2.3%; mean across all lesions and contralateral striatum, respectively). Conversely, the Vv values differed between lesions (Fig. 5; Stroke: 1.1 ± 0.3%; C6 glioma: 3.4 ± 1.2% and 9LGS glioma: 8.2 ± 1.7%) as well as inside each glioma models (Fig. 5). Again, images from all animals (Supp. Figs 6–8) suggest excellent reproducibility.

In stroke, tHct decreased to 19.5 ± 2.5% two hours after stroke onset (p < 0.0001 vs striatum). This reduction was not homogeneous within the region with reduced ADC (from 852 ± 54 µm²/s in healthy striatum to 544 ± 57 µm²/s in the stroke lesion) and part of the reduced ADC region had no reduction in tHct (Fig. 5a, white dashed line and Fig. 5b, black line).

For the two glioma models, the mean reduction in tHct was similar to that of stroke (Hct became 18.5 ± 2.3% in the 9LGS glioma and 16.1 ± 1.2% in the C6 glioma; p < 0.00001 vs striatum). The lesion with the most spatially homogeneous reduction in tHct was the 9LGS, as observed on the maps (Fig. 5a) and highlighted by the histogram (Fig. 5c, red curve). In the C6 tumor, the reduction in tHct was more heterogeneous than in 9LGS with the presence of hotspots (Fig. 5a, white arrow) and lower tHct areas (Fig. 5c, blue curve). The ADC values in these two tumors at this stage of growth were 957 ± 76 µm²/s for the C6 and 862 ± 69 µm²/s for the 9LGS, values above that of contralateral striatum (757 ± 34 and 770 ± 60 µm²/s, respectively (p < 0.0002).

## Discussion

In this study, we report the simultaneous mapping of Vp and Vrbc, and thereby tHct, whereas previous ARG studies determined Vp and Vrbc on separated groups of animals and provided no mean to map tHct. These maps show that tHct can be heterogeneous within a lesion. The mean tHct values observed in various brain structures, as well as the ratio between blood and tissular hematocrit, were in accordance with literature (Supp. Table 3) and were modified as expected by EPO and hemodilution. Using the proposed method, we observed in average a 37% tHct reduction in an acute stroke and in two glioma models, a reduction independent of the change in Vv.

tHct values obtained in liver, muscle, salivary gland and brain varied across organs (from 19 to 29%). For the brain, values were in good agreement with previous reports (tHct = 29 ± 1.3% in our control group vs 29.9% across 5 ARG studies, cf. Supp. Table 3^2,11,16–22^). For the salivary gland, no previous report was found. For the muscle, we observed 25.0 ± 1.7% in our control group, a value between that of Klitzman *et al*. (tHct for striated muscle between 10 and 14%^23^) and that of Everett *et al*. (tHct for the muscle = 32.4%^16^). For the liver, we observed Hct = 19.1 ± 2.9%, a value lower than the one obtained by Everett *et al*. (Hct = 30.5% in male rats^16^). Since albumin may accumulate the liver, tHct may be under-estimated for that organ with albumin-based protocols and as a function of the delay between injection and death. Moreover, muscle and liver perfusion strongly depends on anesthetic conditions. As for Hct values, the ratios between organ Hct and blood Hct varied across organs, between 0.48 (Liver) and 0.72 (Brain) under control conditions. As previously reported, injection of EPO one week prior to imaging led to an increase in blood and organ Hct whereas acute isovolemic hemodilution led to a decrease in bHct and tHct^24^. These experiments demonstrate the sensitivity of the proposed approach. The tHct values obtained in this study for the cortex (29.6 ± 3.2%), striatum (28.8 ± 2.8%), and white matter (28.0 ± 2.6%) were within the range of previously published values (Supp. Table 3), despite the fact that previous studies used different animals to obtain RBCs and plasma volumes. In the brain area that we observed, the dual isotope approach yielded tHct maps with no anatomical contrast. For example, there is no tHct difference between corpus callosum and cortex, while a contrast between these two regions is visible on the Vrbv, Vp, and Vv maps (Fig. 4), in line with previous studies^2^. Further studies using this dual isotope approach are required to evaluate tHct distribution in the entire brain. Overall, these results indicate that the dual isotope approach yields values, image contrasts and responses to Hct challenges in line with that of literature and thereby validate the proposed approach. It could be useful to evaluate and further develop the recently proposed MR approach to map Hct^25^.

The two lesion types used in this study led to a reduction in tHct of about 37%, in line with what had been suggested using other methods^11,26,27^. Overall, this reduction in lesional tHct appears independent of change in ADC or in Vv: in acute stroke, there was a reduction in ADC – as expected – and a normal Vv value whereas, in the two glioma models, ADC was above that of the contralateral area (Fig. 5a) but with different Vv status (normal for the C6 glioma and increased for the 9LGS glioma). Overall, these results suggest that ADC estimates obtained by MR and Vv assessed by a single-isotope autoradiography imaging cannot be used to predict a change in Hct in case of acute stroke or tumor lesion.

In gliomas, the reduction in Hct may arise from several factors: edema may reduce the microvascular lumen^11^, an effect that may be partially reversed under corticosteroids; microvessels may change in diameter and shape^28,29^, increasing local tortuosity; intraluminal adhesion factors may be overexpressed^30^; micro-thrombosis may appear^31^. These different factors may hinder the passage of red blood cell without limiting the flow of plasma. Reduced Hct could thus be a driver for angiogenesis and lead to vessel proliferation. This reduction in Hct is a local anemia, which may add to the systemic anemia that cancer and/or the cancer-therapies may induce. The consequence of this anemia is a reduced oxygenation level that can favor malignancy^32^ and limit the response of tumor to therapy^33^. In stroke, a local reduction of Hct had been suggested based on evaluation of the RBCs visible in histology^27^. This reduction, demonstrated by our study and which appears at least 2 hours after occlusion, may arise from the stroke model itself (the nylon thread may block more RBC than plasma) and/or from cytotoxic edema, namely astrocyte endfeed swelling^34^. This local anemic hypoxia may add to the ischemia and worsen its consequences. Further studies should evaluate whether an acute treatment of edema, such as performed for trauma^35^, can help reoxygenate the tissue and benefit stroke patients^36,37^.

In addition to the pathophysiological consequences, the change in tHct also alters the mapping of perfusion parameters. For CT and perfusion MRI, two clinical approaches based on plasmatic tracers, a reduction in Hct of 10% will lead to a 10% overestimation of relative blood volume or relative blood flow. Similarly, ratios between the blood volume in the tumor and that in the normal appearing white matter, a metric used to evaluate the level of angiogenesis, would be overestimated. In the context of fMRI, a local reduction in tHct suggests that the Blood-Oxygen-Level Dependent (BOLD) response – an increased flow of oxygenated red blood cells – could also be limited. Finally, in the context of tissue oxygen saturation mapping^24,38^, considering a normal tHct whereas tHct is reduced by 20% would lead to a ~12% overestimation of tissue oxygen saturation.

One should mention some limitations to our study. Slight registration errors between MRI and autoradiography may have led to inaccuracy in the delineation of structures or lesions. A point of concern is the potential leakage of BSA during the 15 min delay between its injection and the animal death. An extravasation of BSA would major the Vp and lead to an underestimation of Hct. However, Belayev *et al*. showed that albumin does not extravasate during more than one hour after stroke onset^39^. In tumors, we have previously reported that iron oxide particles do not extravasate during the first 15 min after injection^40^. In addition, further studies could consider particles with a larger hydrodynamic diameter than that of albumin – such as the iron oxide particles used in MRI – to monitor the plasma volume.

In conclusion, the dual isotope approach proposed in this study yields Hct values in line with previous techniques that were using different animals for mapping plasma and RBCs volumes. The Hct maps obtained with the proposed approach respond as expected to both EPO and hemodilution challenges. Using this approach, we were able to highlight a 37% reduction in tHct in a stroke and in two different glioma models and showed that this reduction in tHct can be spatially heterogeneous. This local reduction in Hct aggravates the disease and raises methodological issues in perfusion imaging. Further research should evaluate in a clinical context the impact of this tHct reduction.

## Electronic supplementary material

Supplementary text, Figures, and Tables
