# Supplementary material for: Mapping of brain tissue hematocrit in glioma and acute stroke using a dual autoradiography approach
Source: Sci Rep. 2018 Jun 29;8:9878. doi: 10.1038/s41598-018-28082-w (PMC6026160; doi:10.1038/s41598-018-28082-w)
Supplement: Supplementary file 1 — Supplementary text, Figures, and Tables [file 41598_2018_28082_MOESM1_ESM.pdf]

Mapping of brain tissue hematocrit in glioma and acute stroke using a dual autoradiography approach

A. Broisat[1#], B. Lemasson[2#], M. Ahmadi[1], N. Collomb[3], S. Bacot[1], A. Soubies[1], D. Fagret[1], C. Rémy[2], C. Ghezzi[1], E.L. Barbier[2*]

SUPPLEMENTARY INFORMATION

Supplementary materials and methods

MR Imaging

MRI was conducted with a horizontal bore 4.7 T Biospec animal imager (Bruker Biospin, Ettlingen, Germany) with an actively decoupled cross-coil setup (body coil for radiofrequency transmission and quadrature surface coil for signal reception) and Paravision 5.0.1. After second-order shimming, the following MRI protocol was performed as followed. Anatomical T_2-weighted (T_2w) images were acquired using a turbo spin-echo MRI sequence (repetition time (TR)/echo-time (TE) = 4000/33 ms, 2 averages, 31 slices with a field of view (FOV) = 30x30 mm^2, matrix = 128x128 and voxel size = 234x234x800 μm^3). Acquisition duration was 4 min 17 sec. For animals in Stroke and Glioma-groups, Apparent Diffusion Coefficient (ADC) was mapped using a diffusion-weighted, spin-echo, single-shot echo-planar imaging (EPI; TR/TE = 2200/33 ms, 8 averages, 5 slices with FOV = 30x30 mm^2, matrix = 128x128 and voxel size 234x234x800 μm^3). This sequence was applied 6 times; three without diffusion weighting and three times with diffusion weighting (b = 800 $s.mm^{-2}$) in three orthogonal directions. Acquisition duration was 3 min 31 sec.

Mapping of brain hematocrit.

RBC labeling: First, ^{99m}Tc-labeled red blood cells (^{99m}Tc-RBCs) were prepared with the commercial TechneScan PYP kit (Mallinckrodt, UK) containing 11.9 mg sodium pyrophosphate-decahydrate and 3.36 mg tin chloride-dihydrate. To adjust the commercial procedure to the rat blood, TechneScan PYP was reconstituted with a sterile 0.9% sodium chloride solution (saline solution). Whole blood of donor rats was collected on heparin and 5mL were incubated with the appropriate volume of reconstituted kit containing Sn^{2+} in the form of pyrophosphate (1.1-1.3 µg/mL blood) for 6 min at room temperature (RT) with stirring. After a 5 min centrifugation at 700 g, the supernatant was discarded and the cells were washed with saline solution. Freshly eluted ^{99m}Tc pertechnetate (1.3-1.6 GBq in 1.5 mL saline) from a

$^{99}Mo/^{99m}Tc$ generator was then added to the RBCs and incubated for 5 min at RT with stirring. RBCs were sedimented by centrifugation and then washed by adding 2 ml of saline solution. The cycles of washing were repeated (2 to 4 times), up to obtain less than 2% of radioactivity in the supernatant. All the fractions activities were measured using a dose calibrator Capintec CRC-15R (Aries, United States). Finally, before reinjection to rats, plasma was added to radiolabeled RBCs to enable their storage at 4°C up to 6h. The labeling yield was also calculated. Radiolabeled RBCs *in vitro* stability was evaluated by centrifugation at 6h and *in vivo* stability was also determined by measuring RBC activity following euthanasia.

Plasma labeling: Radiolabeling of Bovine Serum Albumin (BSA) with ^{125}I was prepared as previously described by Salacinski *et al* [1] and modified as follows: 10% BSA (500 µg, Acros Organics) was radiolabeled with ^{125}I (37 MBq, Perkin Elmer, France) in phosphate buffer (50 mM, pH 7.4) using Iodogen-coated tube (50 µg, Pierce®, ThermoScientific, USA). The radiochemical purity (RCP) was determined by thin-layer chromatography on silica gel RP-18 (Merck, Germany) in acetonitrile/ H2O (60:40, v:v) as mobile phase and on ITLC™-SG (Pall Corporation, Port Washington, NY, USA) in physiological serum as mobile phase. It was then analyzed using a ScanRam radioTLC Detector (LabLogic, UK). Furthermore, *in vivo* stability was also determined by measuring plasma activity following euthanasia.

Image analysis: Image analysis was performed using Image-J software [2] (Fig. 1). *Exp2* intensity was corrected to *Exp1* using the internal references containing ^{125}I only (Supp. Fig. 1). Using the ratio between the pure ^{125}I signals obtained at Exp1 and at Exp2, a correction factor was applied to the Exp2 slice. The slices were then co-registered using an automated algorithm [3] and *Exp2* was subtracted from *Exp1* to obtain maps of the distribution of ^{99m}Tc-RBC only (Fig. 1, step 1). Regions of interest were drawn on the three reference tissues (liver, salivary gland and muscle) whose activities had been previously determined using gamma-well counting. A linear regression was performed to determine the relationship between autoradiography signal

intensity and the ^{99m}Tc or ^{125}I activity (MBq/ml). Autoradiographic images of brain slices were then converted into MBq.ml^{-1}, based on the values from reference tissue obtained by GWC (%ID.g^{-1}; Fig. 1, step 2). Red blood cells distribution volume (Vrbc), plasma distribution volume (Vp) and vascular volume (Vv) images were then derived from these concentrations using (Fig. 1, step 3):

[Equation 1] $$Vrbc = \frac{[^{99m}Tc\text{−}RBC]_{tissue} \times bHct}{[^{99m}Tc\text{−}RBC]_{blood}}$$

where $[^{99m}\text{Tc-RBC}]_{\text{tissue}}$ is the concentration of injected ^{99m}Tc-RBC per unit mass of tissue, $[^{99m}\text{Tc-RBC}]_{\text{blood}}$ is the concentration of ^{99m}Tc-RBC in the blood sample as determined by GWC.

[Equation 2] $$Vp = \frac{[^{125}I\text{−}BSA]_{tissue} \times (1 - bHct)}{[^{125}I\text{−}BSA]_{blood}}$$

where $[^{125}\text{I-BSA}]_{\text{tissue}}$ is the concentration of injected ^{125}I-BSA per unit mass of tissue, $[^{125}\text{I-BSA}]_{\text{blood}}$ is the concentration of ^{125}I-BSA in the blood sample as determined by GWC.

[Equation 3] $$Vv = Vrbc + Vp$$

Finally, tissue hematocrit (tHct) was calculated as (Fig. 1, step 4):

[Equation 4] $$tHct = \left(\frac{Vrbc}{Vv} \times 100\right)$$

Supplementary figures and tables

Supp. Figure 1

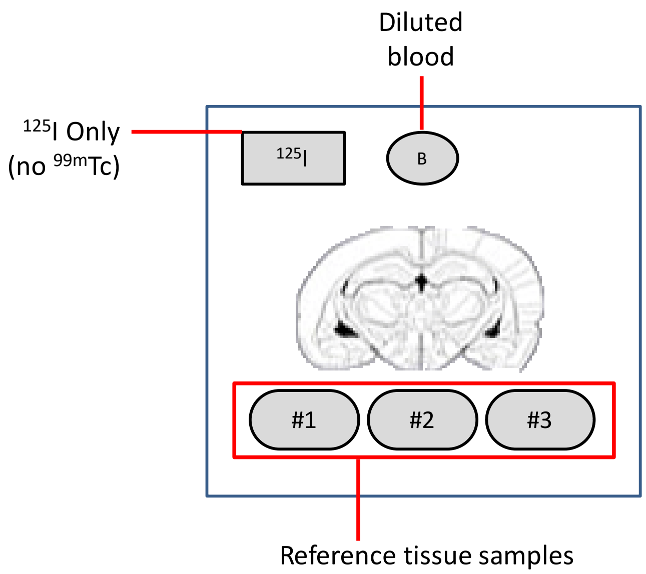

Supp. Figure 1. Schematic of the organization of an autoradiographic slice. The three tissue samples mentioned in the manuscript (liver, salivary gland and muscle) appears as #1, #2, and #3. B corresponds to the diluted blood and ^{125}I corresponds to a drop of pure iodine.

Supp. Figure 2

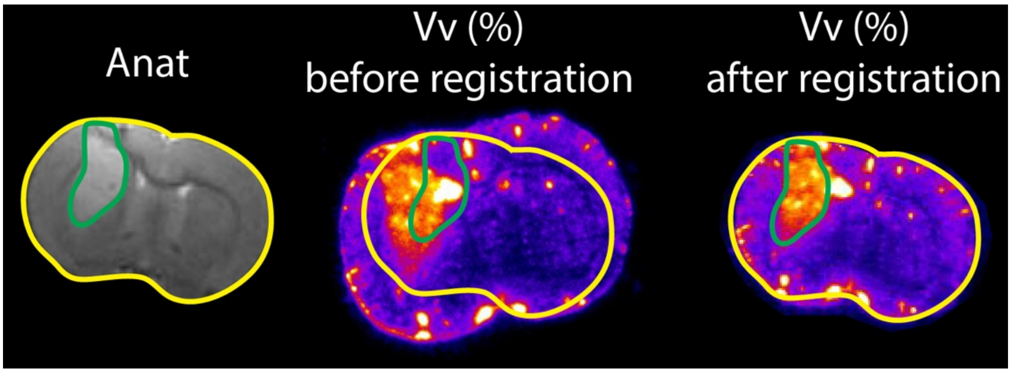

Supp. Figure 2. Example of registration between MRI and autoradiography for a tumor-bearing animal. The yellow line delineates the brain and the green line delineates the tumor. One can observe that the lesion is well depicted in the Vv map after registration and that almost the entire ventricle is well excluded from the lesion

Supp. Figure 3

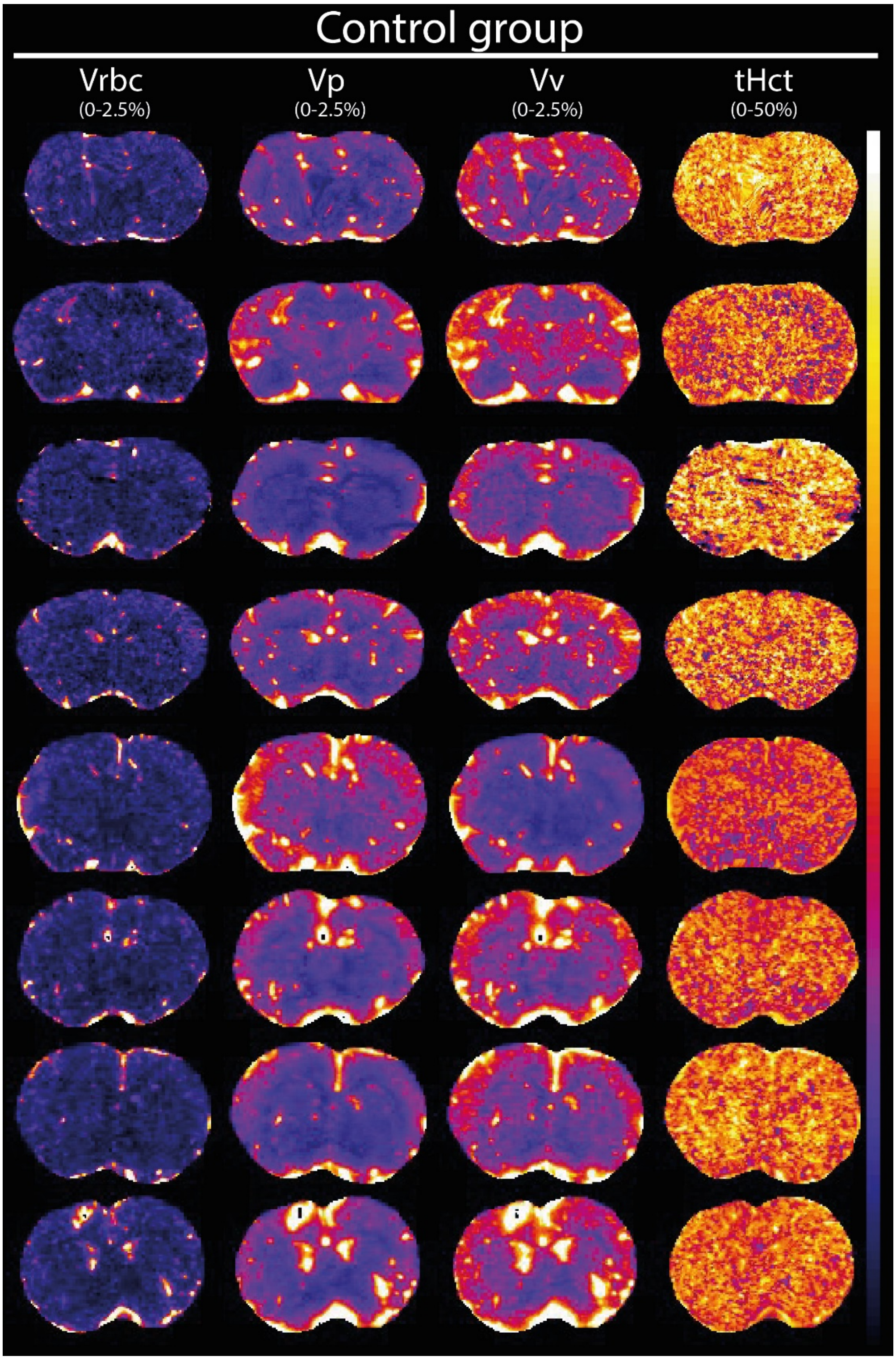

Supp. Figure 3. Maps of red blood cells (Vrbc), plasmatic (Vp), vascular volume (Vv) and tissue hematocrit (tHct) obtained by autoradiography for all animals of the Control group.

Supp. Figure 4

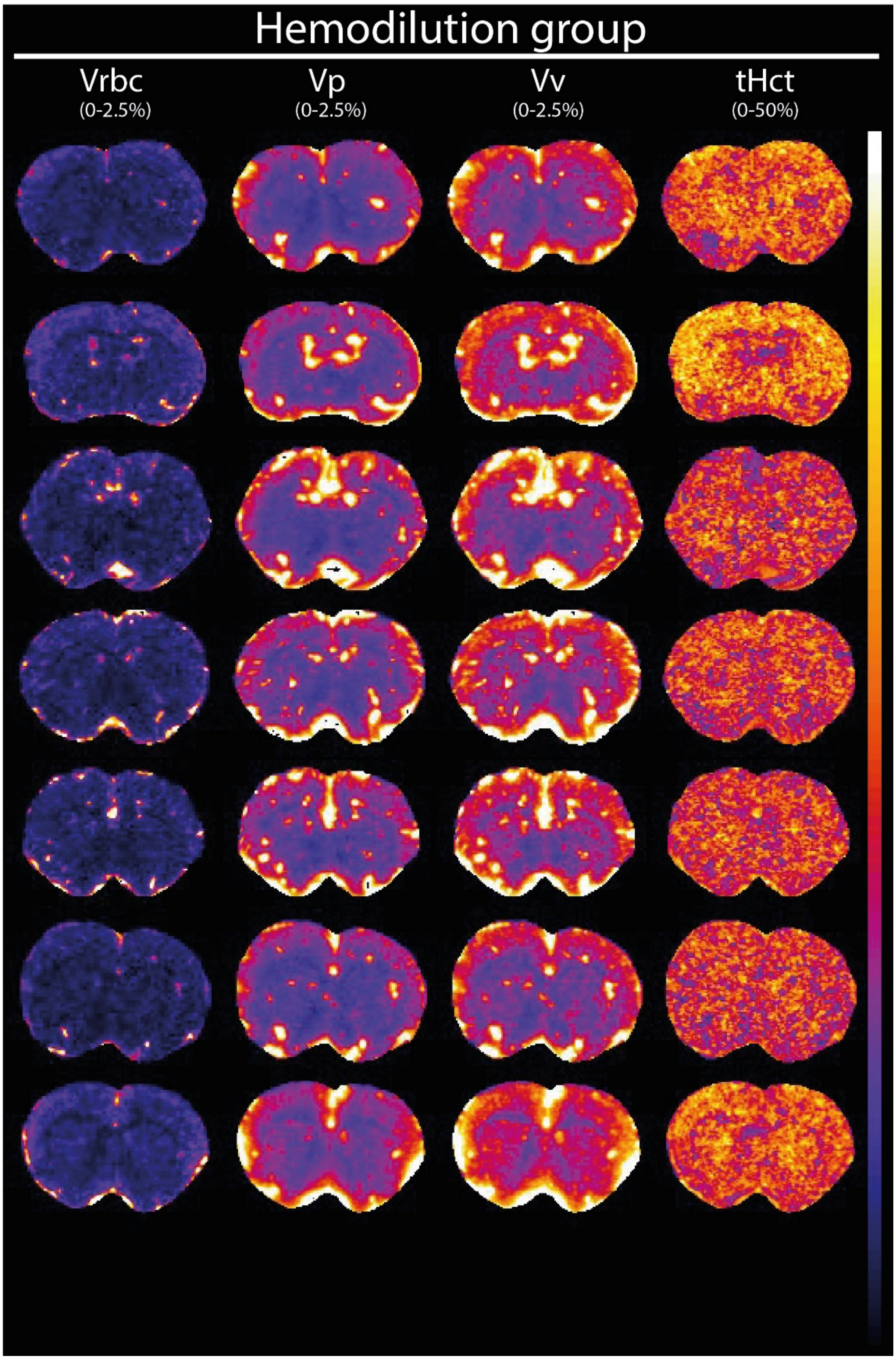

Supp. Figure 4. Maps of red blood cells (Vrbc), plasmatic (Vp), vascular volume (Vv) and tissue hematocrit (tHct) obtained by autoradiography for all animals of the Hemodilution group.

Supp. Figure 5

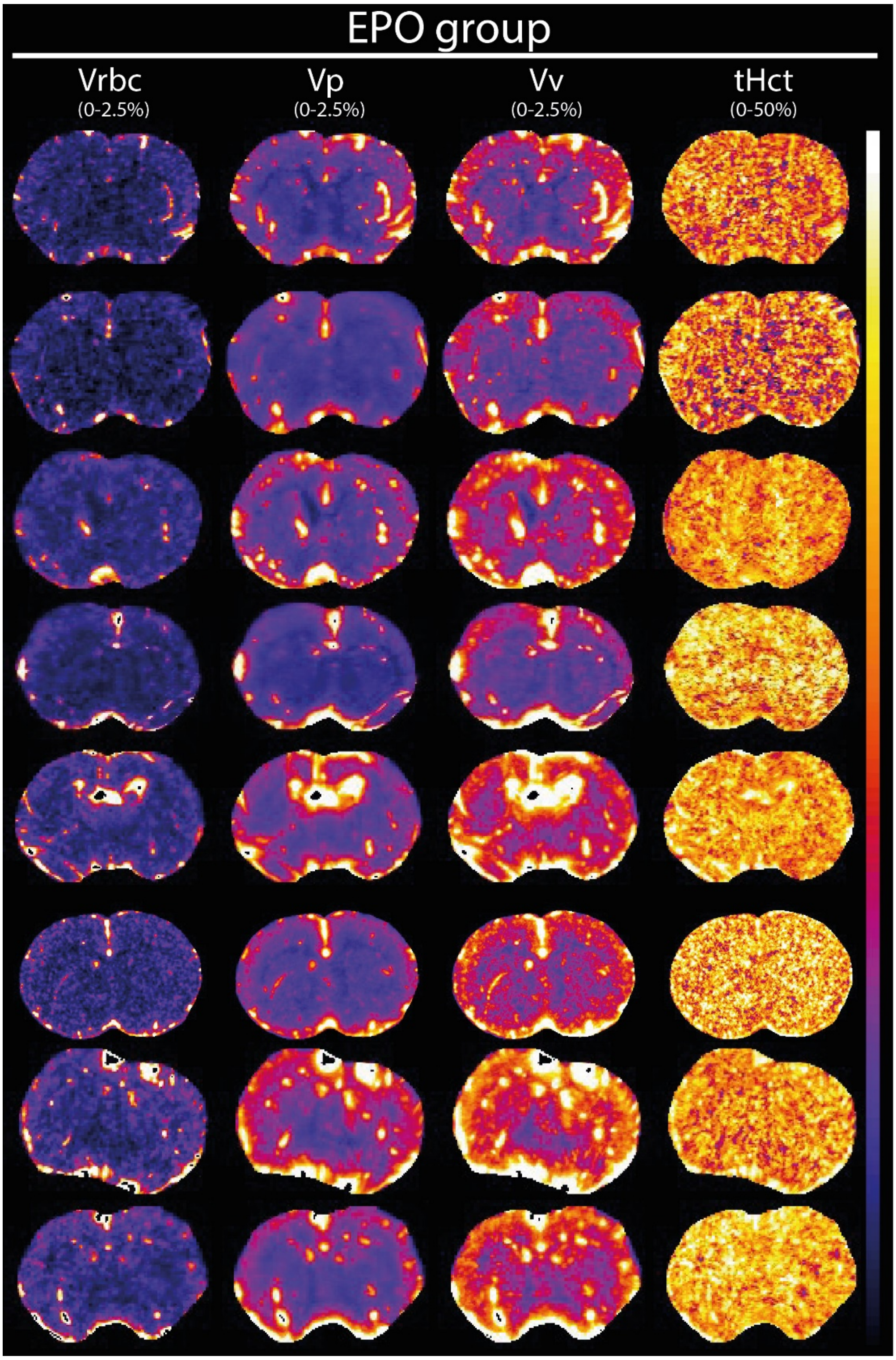

Supp. Figure 5. Maps of red blood cells (Vrbc), plasmatic (Vp), vascular volume (Vv) and tissue hematocrit (tHct) obtained by autoradiography for all animals of the EPO group.

Supp. Figure 6

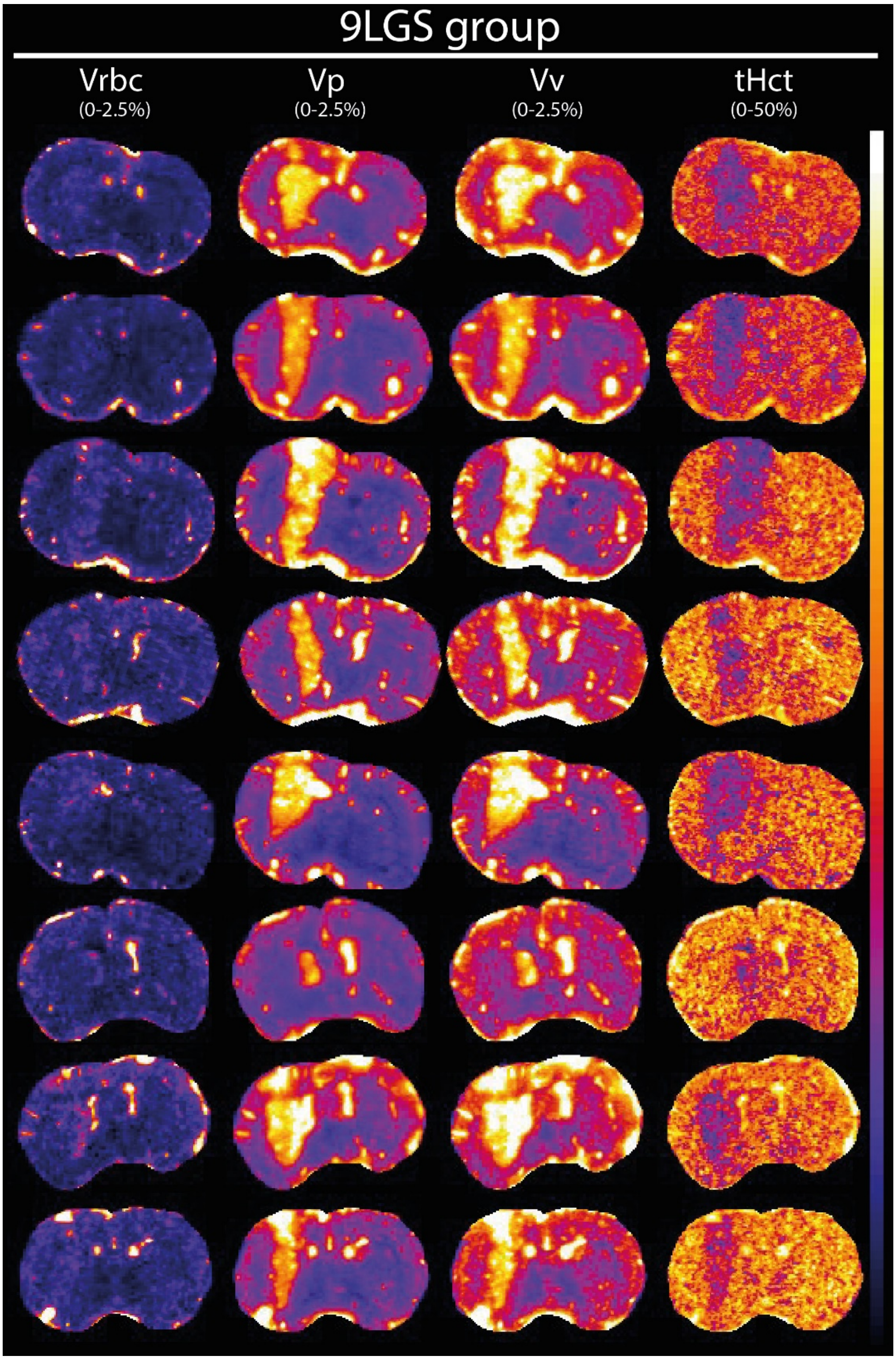

Supp. Figure 6. Maps of red blood cells (Vrbc), plasmatic (Vp), vascular volume (Vv) and tissue hematocrit (tHct) obtained by autoradiography for all animals of the 9LGS group.

Supp. Figure 7

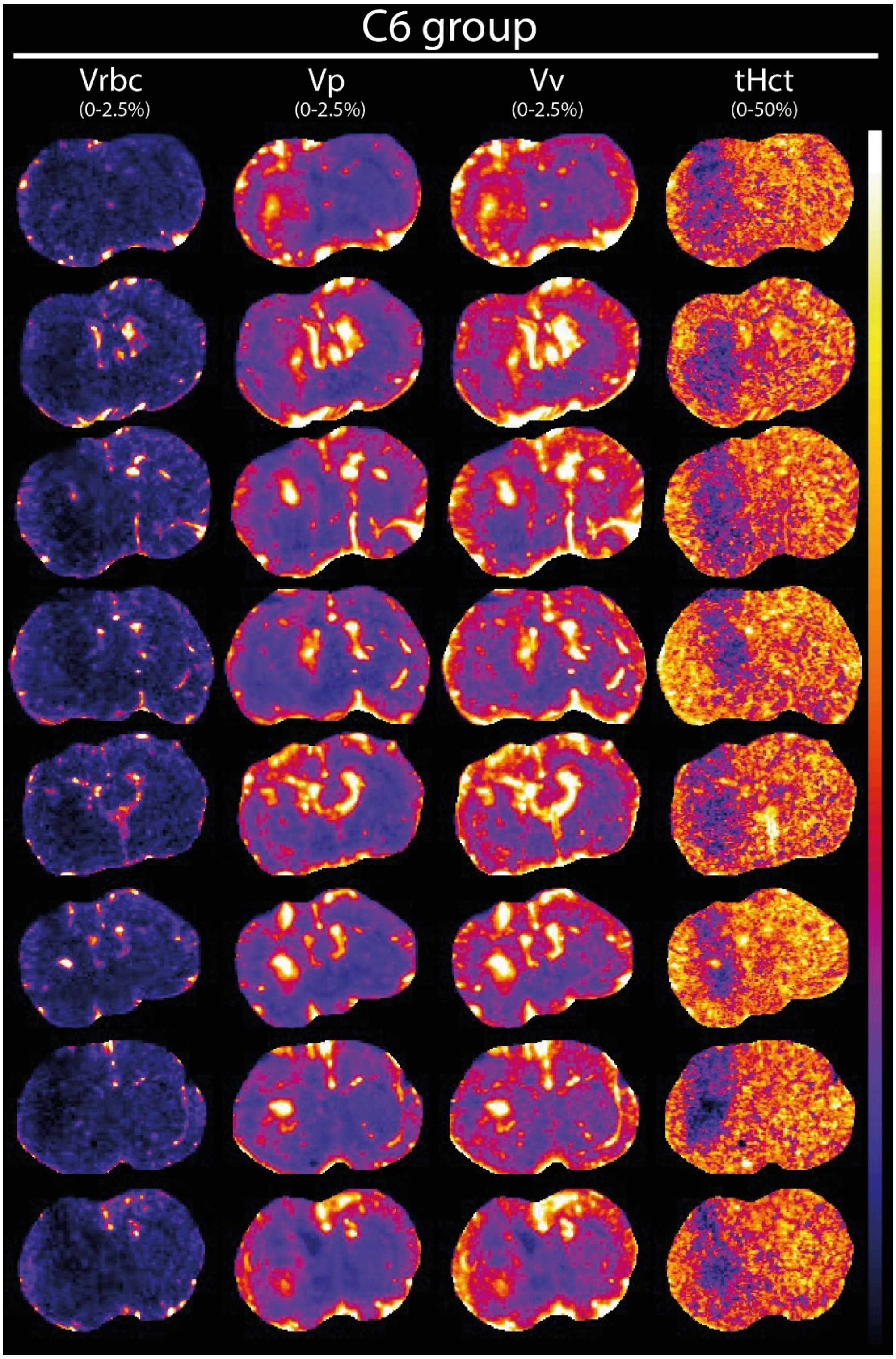

Supp. Figure 7. Maps of red blood cells (Vrbc), plasmatic (Vp), vascular volume (Vv) and tissue hematocrit (tHct) obtained by autoradiography for all animals of the C6 group.

Supp. Figure 8

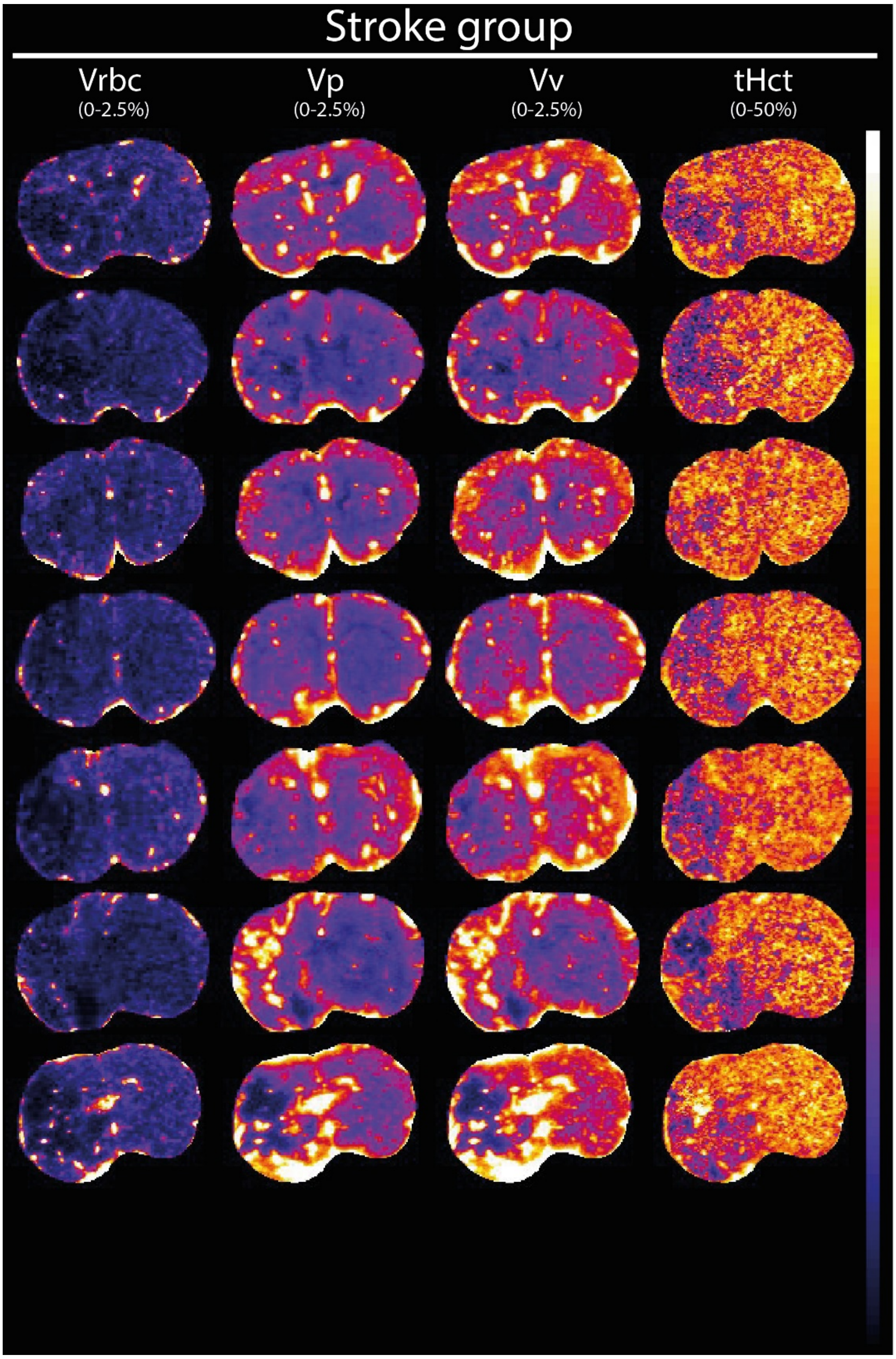

Supp. Figure 8. Maps of red blood cells (Vrbc), plasmatic (Vp), vascular volume (Vv) and tissue hematocrit (tHct) obtained by autoradiography for all animals of the stroke group

	Group	Liver	Muscle	Salivary gl.	Brain	bHct
**tHct or bHct (%)**	**Control**	19.1±2.9	25.0±1.7	19.6±1.2	29.0±1.3	40.1±0.6
	**EPO**	29.1±5.5	34.3±4.5	27.2±2.8	36.7±2.6	48.3±1.9
	**Hemo**	21.7±7.1	22.7±5.7	14.8±2.2	24.1±2.2	31.1±2.1
**Reduction of tHct versus bHct (%)**	**Control**	52.4±7.3	37.6±4.4	51.1±3.3	27.7±2.9	/
	**EPO**	40.2±9.8	29.0±9.4	43.7±5.8	24.0±3.7	/
	**Hemo**	28.4±29.6	25.8±23.1	51.8±9.4	22.0±9.8	/

Supp Table 1: tHct values (%) and percentage of reduction of tHct as compared to bHct across organs. Measurement obtained by GWC. Mean±SD.

	ARG					GWC
	**Group**	**Striatum**	**Cortex**	**WM**	**Brain**	**Brain**
**Mean±SD**	**Control**	28.8±2.8	29.6±3.3	28.0±2.6	28.6±2.8	29.0±1.3
	**EPO**	32.5±3.2	33.9±2.7	31.6±3.2	32.7±3.0	36.7±2.6
	**Hemo**	24.3±1.8	25.4±4.0	22.9±1.9	24.0±2.3	24.2±2.4

Supp Table 2: ARG tHct measured in the Striatum, cortex, white matter (WM) and in the brain.The last column corresponds to the GWC tHct estimates measued in the brain.

Parameter	Everett et al. 1956 [4]	Cremer et al. 1983 [5]	Nakagawa et al 1988 [$] [6]	Bereczki et al. 1992 * [7]	Bereczki et al. 1993 * [8]	Wei et al. 1993 * [9]	**Present study (control group)**	Sakai 1985 [10]	Lammertsma 1984 [11]	Yamauchi 1998 [12]
Whole brain tHct	29.2						**28.6 ± 2.8**	31.3 ± 1.8	28 ± 3	34 ± 2
Cortex tHct		30.9 ± 0.5	32.7 ± 0.8	30 ± 2	28 ± 2	28 ± 1.6	**29.6 ± 3.2**			
Striatum tHct		31.0 ± 0.6	32.7 ± 1.2	36 ± 3	31 ± 2	30 ± 2.2	**28.8 ± 2.8**			
White matter tHct			31.9 ± 1.6	34 ± 4	28 ± 2	30 ± 2.4	**28.0 ± 2.6**			
Hct ratio							**.71**	.76 ± .02	.69	.93 ± .05
**Methods**										
imaging	Post mortem ARG	Post mortem ARG	Post mortem ARG	Post mortem ARG	Post mortem ARG	Post mortem ARG	**Post mortem ARG**	In vivo SPECT	In vivo PET	In vivo PET
tracers	^{131}I-Risa & ^{55}Fe-RBC	^{125}I-Risa & ^{51}Cr-RBC	^{125}I-Risa & ^{51}Cr-RBC	^{125}I-Risa & ^{55}Fe-RBC	^{125}I-Risa & ^{55}Fe-RBC	^{125}I-Risa & ^{55}Fe-RBC	**^{125}I-Risa & ^{99m}Tc-RBC**	^{99m}Tc-RBC & ^{99m}Tc-Alb	^{11}C-RBC & ^{11}C-Alb	^{15}O-RBC & ^{62}Cu-Alb
groups	1 RBC/1 Alb	1 RBC/1 Alb	1 RBC/1 Alb	1 RBC/1 Alb	1 RBC/1 Alb	1 RBC/1 Alb	**1 RBC+Alb**	1 RBC+Alb	1 RBC+Alb	1 RBC+Alb
large vessels inc.	yes	no	no	no	no	no	**no**	yes	yes	yes

($) extrapolated from bar graph

(*) Fenstermacher group

Supp. Table 3: Alb: Albumin; ARG: autoradiography; RBC: Red Blood Cells; large vessels inc.: large vessels included in data analysis

1 Salacinski, P. R., McLean, C., Sykes, J. E., Clement-Jones, V. V. & Lowry, P. J. Iodination of proteins, glycoproteins, and peptides using a solid-phase oxidizing agent, 1,3,4,6-tetrachloro-3 alpha,6 alpha-diphenyl glycoluril (Iodogen). *Anal Biochem* **117**, 136-146 (1981).

2 Schneider, C. A., Rasband, W. S. & Eliceiri, K. W. NIH Image to ImageJ: 25 years of image analysis. *Nat Methods* **9**, 671-675 (2012).

3 Thevenaz, P., Ruttimann, U. E. & Unser, M. A pyramid approach to subpixel registration based on intensity. *IEEE Trans Image Process* **7**, 27-41, doi:10.1109/83.650848 (1998).

4 Everett, N. B., Simmons, B. & Lasher, E. P. Distribution of blood (Fe 59) and plasma (I 131) volumes of rats determined by liquid nitrogen freezing. *Circ Res* **4**, 419-424 (1956).

5 Cremer, J. E. & Seville, M. P. Regional brain blood flow, blood volume, and haematocrit values in the adult rat. *Journal of cerebral blood flow and metabolism : official journal of the International Society of Cerebral Blood Flow and Metabolism* **3**, 254-256, doi:10.1038/jcbfm.1983.35 (1983).

6 Nakagawa, H. *et al.* Dexamethasone effects on vascular volume and tissue hematocrit in experimental RG-2 gliomas and adjacent brain. *J Neurooncol* **6**, 157-168 (1988).

7 Bereczki, D. *et al.* Technique-dependent variations in cerebral microvessel blood volumes and hematocrits in the rat. *J Appl Physiol (1985)* **73**, 918-924 (1992).
8 Bereczki, D. *et al.* Hypoxia increases velocity of blood flow through parenchymal microvascular systems in rat brain. *Journal of cerebral blood flow and metabolism : official journal of the International Society of Cerebral Blood Flow and Metabolism* **13**, 475-486, doi:10.1038/jcbfm.1993.62 (1993).
9 Wei, L. *et al.* The velocities of red cell and plasma flows through parenchymal microvessels of rat brain are decreased by pentobarbital. *Journal of cerebral blood flow and metabolism : official journal of the International Society of Cerebral Blood Flow and Metabolism* **13**, 487-497, doi:10.1038/jcbfm.1993.63 (1993).
10 Sakai, F. *et al.* Regional cerebral blood volume and hematocrit measured in normal human volunteers by single-photon emission computed tomography. *Journal of cerebral blood flow and metabolism : official journal of the International Society of Cerebral Blood Flow and Metabolism* **5**, 207-213, doi:10.1038/jcbfm.1985.27 (1985).
11 Lammertsma, A. A. *et al.* In vivo measurement of regional cerebral haematocrit using positron emission tomography. *Journal of cerebral blood flow and metabolism : official journal of the International Society of Cerebral Blood Flow and Metabolism* **4**, 317-322, doi:10.1038/jcbfm.1984.47 (1984).
12 Yamauchi, H., Fukuyama, H., Nagahama, Y., Katsumi, Y. & Okazawa, H. Cerebral hematocrit decreases with hemodynamic compromise in carotid artery occlusion: a PET study. *Stroke; a journal of cerebral circulation* **29**, 98-103 (1998).
